# Perioperative outcomes of robot-assisted versus laparoscopic liver resection for cavernous hemangioma: a propensity score matching study

**DOI:** 10.1007/s00464-022-09834-2

**Published:** 2023-02-21

**Authors:** Wei Zhang, Junjie Liu, Zunyi Zhang, Yuwei Wang, Shuai Xiang, Lin Chen, Peng Zhu, Wanguang Zhang, Chang Shu, Wan Yee Lau, Bixiang Zhang, Xiaoping Chen

**Affiliations:** 1 Department of Surgery, Hepatic Surgery Center, Tongji Hospital, Tongji Medical College, Huazhong University of Science and Technology, 1095 Jiefang Avenue, Wuhan, 430030 People’s Republic of China; 2 Surgery Administrator Office, Tongji Hospital, Tongji Medical College, Huazhong University of Science and Technology, Wuhan, China; 3 Faculty of Medicine, Prince of Wales Hospital, The Chinese University of Hong Kong, Shatin, Hong Kong SAR China

**Keywords:** Liver hemangioma, Laparoscopic liver resection, Robot-assisted liver resection, Minimally invasive liver surgery

## Abstract

**Background:**

Minimally invasive techniques have increasingly been adopted for liver resection. This study aimed to compare the perioperative outcomes of robot-assisted liver resection (RALR) with laparoscopic liver resection (LLR) for liver cavernous hemangioma and to evaluate the treatment feasibility and safety.

**Methods:**

A retrospective study of prospectively collected data was conducted on consecutive patients who underwent RALR (*n* = 43) and LLR (*n* = 244) for liver cavernous hemangioma between February 2015 and June 2021 at our institution. Patient demographics, tumor characteristics, and intraoperative and postoperative outcomes were analyzed and compared using propensity score matching.

**Results:**

The postoperative hospital stay was significantly shorter (*P* = 0.016) in the RALR group. There were no significant differences between the two groups in overall operative time, intraoperative blood loss, blood transfusion rates, conversion to open surgery or complication rates. There was no perioperative mortality. Multivariate analysis showed that hemangiomas located in posterosuperior liver segments and those in close proximity to major vascular structures were independent predictors of increased intraoperative blood loss (*P* = 0.013 and *P* = 0.001, respectively). For patients with hemangioma in close proximity to major vascular structures, there were no significant differences in perioperative outcomes between the two groups, with the exception that intraoperative blood loss in the RALR group was significantly less than that in the LLR group (350 ml vs. 450 ml, *P* = 0.044).

**Conclusions:**

Both RALR and LLR were safe and feasible for treating liver hemangioma in well-selected patients. For patients with liver hemangioma in close proximity to major vascular structures, RALR was better than conventional laparoscopic surgery in reducing intraoperative blood loss.

Liver cavernous hemangioma is the most common benign liver lesion with an estimated prevalence of 5–20% [1]. These lesions occur most frequently in adult women, and are usually detected between the third and fifth decades of life [2]. Liver hemangioma is usually asymptomatic, and is incidentally diagnosed on imaging studies. Once diagnosed, the majority of these lesions do not require any clinical intervention. Observation of asymptomatic lesions using routine follow-up and imaging is usually adequate [3, 4].

Surgical resection is the only curative treatment. Indications for surgery include the presence of progressive symptoms, spontaneous or traumatic rupture, rapidly enlarging lesions, Kasabach–Merritt syndrome and an unclear diagnosis [5]. Traditional open liver resection for hemangioma requires a long subcostal incision followed by a long postoperative recovery and a high complication rate [6, 7]. Moreover, massive intraoperative hemorrhage remains a major challenge during liver resection or enucleation for hemangiomas [8–10].

Minimally invasive surgery has currently been widely adopted by liver surgeons to treat benign and malignant liver lesions. When compared to open liver resection, minimally invasive resection offers many advantages including decreased intraoperative blood loss, minimized postoperative pain, lower overall morbidity, shorter hospital stay and quicker recovery [11, 12]. In past decades, laparoscopic hepatectomy has gradually been accepted to be safe and feasible, and in selected patients, it is a preferable treatment over open liver resection [13]. However, several inherent limitations have hindered its wide acceptance by liver surgeons, including the limited degree of motion of laparoscopic instruments, the two-dimensional visual field, tremor amplification and poor ergonomics [14]. The introduction of surgical robot has overcome many of the shortcomings of traditional laparoscopic surgery [15] by providing high definition three-dimensional visualization, EndoWrist instruments with 7 degrees of freedom of motion, and tremor filtration. Cohort studies on robot-assisted and/or laparoscopic liver resection for hemangioma have been reported, but most of these studies are small case series or case reports [16–20]. To our knowledge, there have been no studies directly comparing robot-assisted liver resection (RALR) versus laparoscopic liver resection (LLR) for liver hemangiomas.

In this single-center retrospective study, perioperative outcomes of RALR were compared with those of LLR to determine whether robot-assisted surgery was superior to traditional laparoscopic surgery for liver hemangiomas.

## Methods

### Patients

Consecutive patients who underwent minimally invasive liver resection for liver hemangioma at the Hepatic Surgery Center, Tongji Hospital of Huazhong University of Science and Technology, Wuhan, China from February 2015 to June 2021 were included in this study. Based on the minimally invasive surgical technique used, patients were divided into the robot-assisted liver resection group (RALR) and the laparoscopic liver resection group (LLR). The indications for surgery included the presence of progressive abdominal symptoms related to hemangiomas such as upper abdominal discomfort or pain, rapid growth in size and uncertainty of malignancy. Patient information retrieved from the electronic medical records included: patient demographics, preoperative laboratory tests, characteristics of the liver hemangioma, surgery-related variables, postoperative complications, mortality and postsurgical hospital stay. This study was approved by the Institutional Review Board of the hospital and was conducted in accordance with the ethical principles outlined in the Declaration of Helsinki.

### Preoperative evaluation

Preoperative evaluation consisted of a routine panel of blood tests, ultrasonography and contrast-enhanced computed tomography (CT). Magnetic resonance imaging (MRI) was performed if the diagnosis was unclear. For giant hemangiomas adjacent to major vascular structures, three-dimensional reconstruction of the liver vasculature was undertaken. Decisions on surgical treatment for liver hemangioma were made in multidisciplinary meetings attended by liver surgeons, sonographers, radiologists, oncologists, gastroenterologists, and pathologists. The choice between robotic-assisted and laparoscopic techniques was made by the patient and the surgeon. Informed consent was obtained from all patients for the operation and for the data to be used in clinical research.

### Surgical techniques

The robotic-assisted and laparoscopic liver resection techniques used at our institution have been described in previous reports [21, 22]. All procedures were performed by experienced hepatobiliary surgeons who had passed through the learning curves of RALR and LLR. Briefly, the patient was placed in a reversed Trendelenburg position. The semileft lateral decubitus position was employed when the hemangioma was located in the right posterior section or liver segments 6, 7 and 8. The da Vinci S Surgical System (Intuitive Surgical Inc., Sunnyvale, CA) was used for robot-assisted procedures. Five trocars were used: a 12-mm camera port, 2 or 3 working 8-mm robotic ports, and 1 or 2 assistant ports for intraoperative ultrasound, suction, operative instruments and endovascular staplers. The robotic cart was docked at the patient’s head. For laparoscopic surgery, 5 ports (5–12 mm) were placed according to the location of the hemangioma, with trocars distributed around the lesion in a fan-shaped pattern. CO_2_ pneumoperitoneum was established and maintained at 12–14 mmHg. Intraoperative ultrasound (Aloka, Inc., Tokyo, Japan) was routinely performed to identify the relationship between the liver hemangioma and the major blood vessels.

The feeding artery to the liver hemangioma was identified early and controlled with a bull-dog clamp. A urinary catheter was used to encircle the liver pedicle to perform Pringle’s maneuver before liver parenchymal transection. Central venous pressure was controlled below 5 mmHg during liver parenchymal transection. Enucleation was carried out using the technique described previously [23]. For laparoscopic liver resection or enucleation, parenchymal transection was performed by using a harmonic scalpel (Ethicon, Cincinnati, OH, USA). For robot-assisted procedures, a harmonic scalpel was used for transections along straight planes, while the Kelly clamp crushing technique using the endowristed Maryland bipolar forceps (Intuitive Surgical, Sunnyvale, CA, USA) was used for hemangiomas in close proximity to major vascular structures that required a curved transection plane. Divided small diameter vessels were bipolar electrocoagulated, while larger vessels and bile ducts were transected after clamping with Hem-o-loks (Weck Surgical Instruments, Teleflex Medical, Durham, NC, USA) or with Endoscopic Rotating Multiple Clips (Ethicon Endo-Surgery). Laparoscopic linear staplers (EndoGIA, Ethicon Endo-Surgery, Cincinnati, OH, USA) were used to transect the major vascular structures.

After the liver hemangioma was resected, any residual bleeding sites were controlled with suture ligation or electrocautery. The raw surface of the liver was checked for bile leaks. The specimen was inserted into a plastic bag and extracted through a suprapubic transverse incision. A silicone drain was applied to the raw liver transection plane.

### Definitions

The types of hepatectomy were defined according to the Brisbane 2000 terminology [24]. Major hepatectomy was defined as resection of three or more Couinaud liver segments, while minor hepatectomy was defined as resection of fewer than 3 segments [25]. Enucleation was defined as removal of a hemangioma without any loss of adjacent normal hepatic parenchyma. Segments 1, 4a, 7, and 8 were defined as posterosuperior (PS) segments, whereas segments 2, 3, 4b, 5, and 6 were defined as anterolateral segments. Close proximity to major vascular structures was defined as proximity to the porta hepatis, major hepatic veins, or inferior vena cava (IVC). The severity of postoperative complications was graded according to the Clavien‒Dindo classification [26]. Postoperative liver failure and bile leakage were defined according to the International Study Group of Liver Surgery (ISGLS) criteria [27, 28]. Postoperative mortality was defined as death within 90 days of surgery.

### Statistical analysis

Continuous variables are expressed as the median (range). Categorical variables are expressed as numbers with percentages. Statistical analysis was performed using Student’s *t* test for continuous variables and the chi-squared or Fisher exact test for categorical variables. The Mann–Whitney *U* test was used for nonparametric variables. Multivariate logistic regression analyses were performed to identify independent variables associated with increased blood loss. The cutoff level of blood loss was set at the predictive value for red cell transfusion using receiver operating characteristics (ROC) analysis. To reduce confounding effects from heterogeneities between the two groups, propensity score matching (PSM) was performed using a 1:2 ratio based on the nearest neighbor matching method without replacement. Independent variables entered into the propensity model included age, sex, BMI, history of previous upper abdominal operation, size of the largest lesion, the lesion location and its relationship with major blood vessels. Differences were considered significant at *P* values of < 0.05. Statistical analyses were performed using SPSS 22.0 software for Windows.

## Results

### Patient and hemangioma characteristics

During the study period, 287 patients with liver hemangiomas underwent minimally invasive liver resection. There were 43 RALR and 244 LLR. The demographics and preoperative characteristics of the patients are summarized in Table 1. There were 196 female and 91 male patients, with a median age of 48 years (range, 24–66). Fifty-six of the 287 patients were asymptomatic. The most common symptoms were upper abdominal discomfort (*n* = 76), right or left quadrant abdominal pain (*n* = 79) and abdominal mass (*n* = 1). The other important indication for surgery was rapid growth (*n* = 75). The median hemangioma size was 8.6 (range, 5–25) cm. One hundred ninety-two (66.9%) patients had a single hemangioma and 95 (33.1%) patients had multiple hemangiomas. The lesions were located in the right hemilivers in 88 (30.7%) patients, left hemilivers in 170 (59.2%) patients and bilateral hemilivers in 29 (10.1%) patients. Eighty-seven patients had their hemangiomas in the posterosuperior segments, whereas 200 patients had hemangiomas in the anteroperipheral segments. In 107 patients, the liver hemangioma was in close proximity to the major hepatic veins or the inferior vena cava, and in 23 patients, it was in proximity to the porta hepatis.

**Table 1:** Demographics and preoperative characteristics of patients underwent robotic versus laparoscopic hepatectomy

Demographics/characteristics	All patients (*n* = 287)	Unmatched			Matched		
		RALR (*n* = 43)	LLR (*n* = 244)	*P* value	RALR (*n* = 43)	LLR (*n* = 86)	*P* value
Age (mean [year] ± SD)	48 (24–66)	48 (26–62)	48 (24–66)	0.944	48 (26–62)	49 (27–66)	0.517
Female gender	196 (68.3%)	30 (69.8%)	166 (68%)	0.822	30 (69.8%)	60 (69.8%)	1.000
BMI (mean ± SD), kg/m^2^	22.66 (16.9–38.1)	22.4 (18.8–32.9)	22.7 (16.9–32.9)	0.982	22.4 (18.8–32.9)	22.5 (18.3–33)	0.891
Accompany gastrointerestinal disease							
Hepatitis	30 (10.5%)	4 (9.3%)	26 (10.7%)	0.789	4 (9.3%)	11 (12.8%)	0.560
Fatty liver disease	33 (11.5%)	6 (14%)	27 (11.1%)	0.773	6 (14%)	10 (11.6%)	0.706
Disease of biliary system	30 (10.5%)	5 (11.6%)	25 (10.2%)	0.998	5 (11.6%)	10 (11.6%)	1.000
Previous upper abdominal operation	3 (2.4%)	2 (4.7%)	5 (2%)	0.629	2 (4.7%)	3 (3.5%)	1.000
Mean preoperative laboratory results							
Hemoglobin (g/L)	128.7 ± 17.6	130.0 ± 19.4	128.4 ± 17.2	0.582	130.0 ± 19.4	128.3 ± 17.9	0.611
Platelet (× 10^9^ /L)	211.0 ± 60.5	218.7 ± 62.6	209.7 ± 60.1	0.367	218.7 ± 62.6	217.0 ± 67.5	0.891
Prothrombin time (s)	13.4 ± 0.7	13.4 ± 0.6	13.4 ± 0.8	0.616	13.4 ± 0.6	13.5 ± 0.7	0.472
Bilirubin (mmol/L)	11.1 ± 5.4	11.1 ± 4.3	11.1 ± 5.6	0.952	11.1 ± 4.3	11.3 ± 5.9	0.898
Albumin (g/L)	41.6 ± 3.8	41.3 ± 3.9	41.7 ± 3.8	0.555	41.3 ± 3.9	41.7 ± 4.9	0.549
Creatinine (mmol/L)	65.1 ± 14.5	65.8 ± 18.3	65.0 ± 13.8	0.752	65.8 ± 18.3	62.6 ± 12.4	0.443
Size of the largest lesion, cm	8.6 (5–25)	9 (5.6–20)	8.5 (5–25)	0.400	9 (5.6–20)	9(5–25)	0.940
< 10 cm	171 (59.6%)	24 (55.8%)	147 (60.2%)	0.756	24 (55.8%)	48(55.8%)	0.764
10–15 cm	107 (37.3%)	17 (39.5%)	90 (36.9%)		17 (39.5%)	36(41.9%)	
> 15 cm	9 (3.1%)	2 (4.7%)	7 (2.9%)		2 (4.7%)	2(2.3%)	
Number of lesions							
Single	192 (66.9%)	35 (81.4%)	157 (64.3%)	**0.028**	35 (81.4%)	62 (72.1%)	0.249
Multiple	95 (33.1%)	8 (18.6%)	87 (35.7%)		8 (18.6%)	24 (27.9%)	
Location							
Right liver	88 (30.7%)	17 (39.5%)	71 (29.1%)	0.389	17 (39.5%)	21 (24.4%)	0.204
Left liver	170 (59.2%)	22 (51.2%)	148 (60.7%)		22 (51.2%)	54 (62.8%)	
Bilateral	29 (10.1%)	4 (9.3%)	25 (10.2%)		4 (9.3%)	11 (12.8%)	
Posterosuperior segments	87 (30.3%)	12 (27.9%)	75 (30.7%)	0.710	12 (27.9%)	25 (29.1%)	0.891
Anteroperipheral segments	200 (69.7%)	31 (72.1%)	169 (69.3%)		31 (72.1%)	61 (70.9%)	
Relationship with major vascular structures							
No relationship	157 (54.7%)	16 (37.2%)	141 (57.8%)	**0.016**	16 (37.2%)	31 (36%)	0.911
Proximity to major hepatic vein or inferior vena cava	107 (37.3%)	20 (46.5%)	87 (35.7%)		20 (46.5%)	43 (50%)	
Proximity to the main portal pedicle	23 (8%)	7 (16.3%)	16 (6.6%)		7 (16.3%)	12 (14%)	

Significant values are given in bold (*p* < 0.05)

### Perioperative outcomes

There were no significant differences in the patient demographics and preoperative characteristics between the RALR and LLR groups, except that the RALR group had significantly higher proportions of solitary lesion (*P* = 0.028) and lesions in close proximity to major vascular structures (*P* = 0.016) than the laparoscopic group (Table 1). After propensity score matching, these imbalances between groups were eliminated.

The data about the surgical procedures, and intraoperative and postoperative outcomes are outlined in Table 2. There were no significant differences between the two groups in the extent or types of liver resection, blood transfusion rates and postoperative laboratory results. The operative time was significantly longer in the RALR group (270 min versus 210 min; *P* = 0.002), whereas the median blood loss was comparable between groups (200 mL versus 200 mL; *P* = 0.579). In the propensity-matched cohorts, there were no significant differences in inflow occlusion time, blood loss, blood transfusion rates or postoperative laboratory results between groups. There was a trend toward a longer operative time in RALR than LLR (270 min vs. 240 min, *P* = 0.062).

**Table 2:** Surgical procedure, intraoperative, and postoperative outcomes of the patients underwent robotic versus laparoscopic hepatectomy

Perioperative outcomes	All patients (*n* = 287)	Unmatched			Matched		
		RALR (*n* = 43)	LLR (*n* = 244)	*P* value	RALR (*n* = 43)	LLR (*n* = 86)	*P* value
Resection extent				0.849			0.560
Major	57 (19.9%)	9 (20.9%)	48 (19.7%)		9 (20.9%)	22 (25.6%)	
Minor	230 (80.1%)	34 (79.1%)	196 (80.3%)		34 (79.1%)	64 (74.4%)	
Types of resection				0.645			0.765
Left lateral sectionectomy	114 (39.7%)	16 (37.2%)	98 (40.2%)		16 (37.2%)	27 (31.4%)	
Left hepatectomy	18 (6.3%)	4 (9.3%)	14 (5.7%)		4 (9.3%)	9 (10.5%)	
Right hepatectomy	9 (3.1%)	0 (0)	9(3.7%)		0 (0)	3 (3.5%)	
Enucleation involving one or two segments	106 (36.9%)	17 (39.5%)	89 (36.5%)		17 (39.5%)	35 (40.7%)	
Enucleation involving ≥ 3 segments	40 (13.9%)	6 (14%)	34 (13.9%)		6 (14%)	12 (14%)	
Operative time (min)	210 (120–480)	270 (120–480)	210 (120–480)	**0.002**	270 (120–480)	240 (120–480)	0.062
Pringle maneuver							
No. (%) of patients	143 (49.8%)	28 (65.1%)	115 (47.1%)	**0.030**	28 (65.1%)	43 (50%)	0.104
Duration (min)	27 (4–97)	33.5 (12–97)	25 (4–90)	0.086	33.5 (12–97)	30 (4–90)	0.339
Blood loss (mL)	200 (50–2500)	200 (50–1500)	200 (50–2500)	0.579	200 (50–1500)	200 (50–2500)	0.418
Blood Transfusion							
No. (%) of patients	34 (11.8%)	3 (7%)	31 (12.7%)	0.284	3 (7%)	14 (16.3%)	0.141
Mean no. of units	4 (2–8)	4 (3–8)	4 (2–8)	0.731	4 (3–8)	4 (2–8)	0.676
Conversion to open	23 (8%)	2 (4.7%)	21 (8.6%)	0.564	2 (4.7%)	9 (10.5%)	0.265
Postoperative laboratory results							
TB (μmol/L), POD 1	13.4 (3.2–71.5)	11.2 (5.1–28.4)	13.6 (3.2–71.5)	0.534	11.2 (5.1–28.4)	13.9 (3.2–71.5)	0.364
POD 7	9.6 (3.4–109.3)	9.5 (4–21)	9.6 (3.4–109.3)	0.437	9.5 (4–21)	9.25 (3.9–109.3)	0.498
Peak	15.4 (4–187.6)	15.7 (5.1–28.5)	15.4 (4–187.6)	0.940	15.7 (5.1–28.5)	17 (5.2–187.6)	0.291
AST(U/L), POD 1	197 (35–2368)	145 (50–1325)	201 (35–2368)	0.398	145 (50–1325)	230 (40–2368)	0.272
POD 7	17 (10–109)	18 (11–84)	17 (10–109)	0.373	18 (11–84)	18 (10–109)	0.722
Peak	199 (35–2368)	168 (50–1325)	201 (35–2368)	0.53	168 (50–1325)	230 (40–2368)	0.346
ALT(U/L), POD 1	170 (32–2153)	153 (32–1205)	172 (32–2153)	0.667	153 (32–1205)	189 (36–2153)	0.660
POD 7	42 (11–262)	51 (11–256)	41 (11–262)	0.21	51 (11–256)	45 (13–262)	0.276
Peak	172 (32–2153)	169 (32–1205)	172 (32–2153)	0.901	169 (32–1205)	189 (36–2153)	0.816
Complication	51 (17.8%)	5 (11.6%)	46 (18.9%)	0.253	5 (11.6%)	20 (23.3%)	0.115
Wound infection	1 (0.3%)	0	1 (0.4%)		0	1 (1.2%)	
Postoperative hemorrhage	1 (0.3%)	0	1 (0.4%)		0	0	
Bile leak	3 (1.0%)	1 (2.3%)	2 (0.8%)		1 (2.3%)	0	
Pleural effusion	41 (14.3%)	4 (9.3%)	37 (15.2%)		4 (9.3%)	16 (18.6%)	
Liver failure	1 (0.3%)	0	1 (0.4%)		0	1 (1.2%)	
Calf deep vein thrombosis	2 (0.7%)	0	2 (0.8%)		0	1 (1.2%)	
Abdominal Abscess	1 (0.3%)	0	1 (0.4%)		0	1 (1.2%)	
Postoperative biliary obstruction	1 (0.3%)	0	1 (0.4%)		0	0	
Clavien–Dindo classification				0.315			0.272
Grade I	42 (14.6%)	5 (11.6%)	36 (14.8%)		5 (11.6%)	14 (16.3%)	
Grade II	11 (3.8%)	0	6 (2.5%)		0	4 (4.7%)	
Grade III	4 (1.4%)	0	4 (1.6%)		0	2 (23%)	
Postsurgical hospital stay (days)	7 (4–29)	7 (4–13)	7 (4–29)	**0.035**	7 (4–13)	7.5 (4–29)	**0.016**
30-day mortality	0	0	0	–	0	0	–

Significant values are given in bold (*p* < 0.05)

Of the 43 patients in the RALR group, only 2 (4.7%) required conversion to open surgery. Although the conversion rate in the LLR group was higher, the difference did not reach significance (8.6% vs. 4.7%, *P* = 0.564). The reasons for conversion to open surgery included intraoperative bleeding in 19 patients, severe adhesions in 3 patients, and hemodynamic instability after Pringle’s maneuver in 1 patient. After propensity score matching, the conversion rate in LLR was still higher, but it again failed to reach a significant difference (10.5% vs. 4.7%, *P* = 0.265).

The complication rate was 11.6% (5 of 43 patients) in the RALR group, and 18.9% (46 of 244 patients) in the LLR group (*P* = 0.253). All 5 complications in the RALR group were grade I. Four patients (1.6%) in the LLR group developed grade III complications. The most common complication was pleural effusion, which occurred in 4 (9.3%) patients in the RALR group and 37(15.2%) patients in the LLR group. There was no perioperative mortality in either group. The postoperative hospital stay was significantly shorter in the RALR group than in the LLR group (*P* = 0.035). After propensity score matching, the complication rates showed no significant difference between groups (11.6% in RALR vs. 23.3% in LLR, *P* = 0.115). Patients who underwent RALR had a significantly shorter postsurgical hospital stay than patients who underwent LLR (*P* = 0.016).

### Factors associated with increased intraoperative blood loss

The mean and median blood losses were 324.5 mL and 200 (range, 200–2500) mL, respectively. ROC analysis showed that the predictive value of blood loss in patients who received red cell transfusion was 550 ml. On univariate analysis, increased blood loss was significantly correlated with hemangiomas located in posterosuperior segments, in close proximity to major vascular structures, lesions > 10 cm, lesions in right/bilateral hemilivers, major liver resection, Pringle’s maneuver, conversion to open surgery, complication rate, operation time and postoperative hospital stay (Table 3). On multivariate analysis, hemangiomas located in posterosuperior segments and in close proximity to major vascular structures were independent predictors of increased intraoperative blood loss (*P* = 0.013 and *P* = 0.001, respectively; Table 4).

**Table 3:** Univariate analysis of associations between intraoperative blood loss and various parameters

Variable	≤ 550 ml (*n* = 236)	> 550 ml (*n* = 51)	*P* value
Gender			
Male	74 (31.4%)	17 (33.3%)	0.783
Female	162 (68.6%)	34 (66.7%)	
Age			
≤ 48	127 (53.8%)	28 (54.9%)	0.888
> 48	109 (46.2%)	23 (45.1%)	
BMI			
< 24	159 (67.4%)	34 (66.7%)	0.922
≥ 24	77 (32.6%)	17 (33.3%)	
Underlying chronic hepatitis			
Presence	25 (10.6%)	5 (9.8%)	0.867
Absence	211 (89.4%)	46 (90.2%)	
Fatty liver disease			
Presence	27 (11.4%)	6 (11.8%)	0.948
Absence	209 (88.6%)	45 (88.5%)	
Size (cm)			
< 10	158	13	** < 0.001**
≥ 10	78	38	
Location 1			
Left	159 (67.4%)	11(21.6%)	** < 0.001**
Right	58 (24.6%)	30(58.8%)	
Bilateral	19 (8.1%)	10(19.6%)	
Location 2			
Posterosuperior segments	48 (20.3%)	39 (76.5%)	** < 0.001**
Anteroperipheral segments	188 (79.7%)	12 (23.5%)	
Relationship with major blood vessels			
No relationship	156 (66.1%)	1 (2%)	** < 0.001**
Proximity to major hepatic vein or inferior vena cava	61 (25.8%)	46 (90.2%)	
Proximity to the first hilum	19 (8.1%)	4 (7.8%)	
Platelet (× 10^9^/L)			
≤ 206	119 (50.4%)	26 (51%)	0.943
> 206	117 (49.6%)	25 (49%)	
Prothrombin time (s)			
≤ 13.4	126 (53.4%)	31 (60.8%)	0.336
> 13.4	110 (46.6%)	20(39.2%)	
Resection extent			
Major	30 (12.7%)	27 (52.9%)	** < 0.001**
Minor	206 (87.3%)	24 (47.1%	
Pringle maneuver			
Presence	100 (42.4%)	43 (84.3%)	** < 0.001**
Absence	136 (57.6%)	8 (15.7%)	
Conversion to open			
Presence	3 (1.3%)	20 (39.2%)	** < 0.001**
Absence	233 (98.7%)	31 (60.8%)	
Operative time (min)			
≤ 210	147 (62.3%)	2 (3.9%)	** < 0.001**
> 210	89 (37.7%)	49 (96.1%)	
Operative method			
Robotic	39 (16.5%)	4 (7.8%)	0.115
Laparoscopic	197 (83.5%)	47 (92.2%)	
Complication			
Presence	34 (14.4%)	23 (45.1%)	** < 0.001**
Absence	202 (85.6%)	28 (54.9%)	
Postsurgical hospital stay (days)			
≤ 7	140 (59.3%)	19 (37.3%)	**0.004**
> 7	96 (40.7%)	32 (62.7%)	

Significant values are given in bold (*p* < 0.05)

**Table 4:** Multivariate analysis of associations between intraoperative blood loss and various preoperative parameters

Variables	Odds ratio	95% Confidence Interval	*P* value
Hemangioma size			
≥ 10 cm, < 10 cm	2.286	0.982–5.323	0.055
Hemangioma location 1			
Right/Bilateral, Left,	1.638	0.44–6.094	0.462
Hemangioma location 2			
Posterosuperior, Anteroperipheral	5.054	1.416–18.031	**0.013**
Proximity to major vascular structures			
Yes, No	33.440	4.13–270.652	**0.001**
Resection extent			
Major, Minor	2.027	0.883–4.655	0.096

Significant values are given in bold (*p* < 0.05)

### RALR versus LLR for patients with hemangioma in close proximity to major vascular structures

For patients with hemangioma in close proximity to major vascular structures, the RALR and LLR groups were comparable in patient demographics and hemangioma characteristics. There were no significant differences in operative time, Pringle’s maneuver, conversion rate, complication rate or postoperative hospital stay. The intraoperative blood loss in the RALR group was significantly less than that in the LLR group (350 ml vs. 500 ml, *P* = 0.018) with significantly fewer patients requiring blood transfusion in the RALR group (11.1% vs. 30.1%, *P* = 0.046).

After propensity score matching, the two groups showed no significant difference in baseline characteristics. No significant differences were observed in Pringle’s maneuver, blood transfusion, conversion rate, complication rate or postoperative hospital stay between groups. Although the RALR group required a longer operative time (330 min vs. 300 min), the difference did not reach significance (*P* = 0.06). Compared to the LLR group, the RALR group had significantly less intraoperative blood loss (350 ml vs. 450 ml, *P* = 0.044) (Table 5).

**Table 5:** Comparison of perioperative outcomes of robotic vs laparoscopic hepatectomy for patients with hemangioma in close proximity to major vascular structures

Demographics/Characteristics	All patients (*n* = 130)	Unmatched			Matched		
		RALR (*n* = 27)	LLR (*n* = 103)	*P* value	RALR (*n* = 27)	LLR (*n* = 54)	P value
Age (mean [year] ± SD)	48 (26–66)	48 (26–62)	48 (29–66)	0.266	48 (26–62)	48 (29–66)	0.330
Female gender	97 (74.6%)	20 (74.1%)	77 (74.8%)	0.942	20 (74.1%)	37 (68.5%)	0.606
BMI (mean ± SD), kg/m^2^	22.5 (17.9–31.6)	22.4 (18.8–31.6)	22.6 (17.9–30.5)	0.991	22.4 (18.8–31.6)	22.7 (18.2–30.5)	0.865
Accompany gastrointerestinal disease							
Hepatitis	14 (10.8%)	2 (7.4%)	12 (11.7%)	0.527	2 (7.4%)	7 (13%)	0.708
Fatty liver disease	17 (13.1%)	5 (18.5%)	12 (11.7%)	0.534	5 (18.5%)	8 (14.8%)	0.915
Disease of biliary system	14 (10.8%)	3 (11.1%)	10 (9.7%)	1.000	3 (11.1%)	5 (9.3%)	1.000
Previous upper abdominal operation	4 (3.1%)	1 (3.7%)	3 (2.9%)	1.000	1 (3.7%)	2 (3.7%)	1.000
Size of the largest lesion, cm	10 (5–25)	9.1 (5.6–20)	10 (5–25)	0.243	9.1 (5.6–20)	9.8 (5–25)	0.980
< 10 cm	56 (43.1%)	14 (51.9%)	42 (40.8%)	0.547	14 (51.9%)	27 (50%)	0.421
10–15 cm	65 (50%)	11 (40.7%)	54 (52.4%)		11 (40.7%)	26 (48.1%)	
> 15 cm	9 (6.9%)	2 (7.4%)	7 (6.8%)		2 (7.4%)	1 (1.9%)	
Location							
Right liver	67 (51.5%)	16 (59.3%)	51 (49.5%)	0.648	16 (59.3%)	22 (40.7%)	0.231
Left liver	38 (29.2%)	7 (25.9%)	31 (30.1%)		7 (25.9%)	24 (44.4%)	
Bilateral	25 (19.2%)	4 (14.8%)	21 (20.4%)		4 (14.8%)	8 (14.8%)	
Posterosuperior segments	77 (59.2%)	12 (44.4%)	65 (63.1%)	0.079	12 (44.4%)	22 (40.7%)	0.750
Anteroperipheral segments	53 (40.8%)	15 (55.6%)	38 (36.9%)		15 (55.6%)	32 (59.3%)	
Major resection	53 (40.8%)	8 (29.6%)	45 (43.7%)	0.186	8 (29.6%)	20 (37%)	0.509
Types of resection				0.340			0.758
Left lateral sectionectomy	7 (5.4%)	2 (7.4%)	5 (4.9%)		2 (7.4%)	3 (5.6%)	
Left hepatectomy	15 (11.5%)	4 (14.8%)	11 (10.7%)		4 (14.8%)	10 (18.5%)	
Right hepatectomy	9 (6.9%)	0 (0)	9 (8.7%)		0 (0)	3 (5.6%)	
Enucleation involving one or two segments	64 (49.2%)	16 (59.3%)	48 (46.6%)		16 (59.3%)	29 (53.7%)	
Enucleation involving ≥ 3 segments	35 (26.9%)	5 (18.5%)	30 (29.1%)		5 (18.5%)	9 (16.7%)	
Operative time (min)	330 (120–480)	330 (120–480)	330 (120–480)	0.141	330 (120–480)	300 (150–480)	0.060
Pringle maneuver							
No. (%) of patients	100 (76.9%)	24 (88.9%)	76 (73.8%)	0.097	24 (88.9%)	38 (70.4%)	0.164
Duration (min)	30 (4–97)	45 (12–97)	30 (4–90)	0.095	45 (12–97)	25 (4–80)	0.081
Blood loss (mL)	400 (50–2500)	350 (50–1500)	500 (50–2500)	**0.018**	350 (50–1500)	450 (50–2500)	**0.044**
Blood Transfusion							
No. (%) of patients	34 (26.2%)	3 (11.1%)	31 (30.1%)	**0.046**	3 (11.1%)	13 (24.1%)	0.167
Mean No. of units	4 (2–8)	4 (3–8)	4 (2–8)	0.731	4 (3–8)	4 (2–8)	0.704
Conversion, No.(%)	23 (17.7%)	2 (7.4%)	21 (20.4%)	0.197	2 (7.4%)	10 (18.5%)	0.320
Complication, No.(%)	30 (23.1%)	5 (18.5%)	25 (24.3%)	0.528	5 (18.5%)	13 (24.1%)	0.571
Postsurgical hospital stay (days)	8 (4–29)	7 (4–13)	8 (4–29)	0.095	7 (4–13)	8 (4–29)	0.113

Significant values are given in bold (*p* < 0.05)

## Discussion

Cavernous hemangioma represents the most common benign lesion of the liver [1]. Most hemangiomas do not require any treatment, as the natural history of liver hemangioma is generally uncomplicated. Surgery should only be considered for patients with complicated or symptomatic lesions, or when malignancy cannot be excluded [5, 7]. Surgical treatment is the only effective treatment for liver hemangiomas. Although minimally invasive liver resection has been increasingly adopted for many benign or malignant liver lesions [29], there has been no consensus on whether RALR can provide better treatment outcomes than conventional LLR.

Shin et al. [30] reported that laparoscopic liver resection for liver hemangioma produced better perioperative outcomes than open surgery. A recently reported study using PSM analysis showed similar findings [31]. However, other studies revealed no significant differences in operative time, estimated blood loss, or major morbidity and mortality rates between laparoscopic and open surgery [32, 33]. A small study comparing the perioperative outcomes of robot-assisted, laparoscopic and open hemihepatectomy for giant hemangiomas over 10 cm [34] showed robotic hemihepatectomy to be associated with significantly less intraoperative blood loss and a shorter operative time and postoperative hospital stay than laparoscopic surgery.

The results of our study showed that both robot-assisted and laparoscopic liver resection were feasible, safe and effective in treating large to giant hemangiomas. There were no significant differences in blood loss, conversion or postoperative overall morbidity rates between RALR and LLR. However, RALR had a longer operative time but a shorter postsurgical hospital stay. These findings are consistent with the results obtained in a previous study and a recent meta-analyses [35, 36]. As robot-assisted liver resection has only recently been adopted by liver surgeons, the learning curve can be an important factor contributing to the differences in operation time reported by different surgeons. Tsung et al. [37] showed the operation time was significantly decreased as experience with robotic technology accumulated. Furthermore, in robotic surgery, extra time is required for changing instruments, and docking and undocking the robotic system as stated by the International Consensus Statement on robotic hepatectomy in 2018 [12].

Liver hemangioma is a benign disease. Enucleation is the preferred surgical treatment as it avoids unnecessary loss of healthy liver parenchyma [38–40]. The identification of a plane between the pseudocapsule of a hemangioma and liver parenchyma is the most challenging part of the procedure. Delicate detachment of the lesion from the pseudocapsule carries a risk of bleeding, especially when the hemangioma is adjacent to major vascular structures. Our study showed robotic surgery to have a significant advantage in reducing intraoperative blood loss over traditional laparoscopic surgery for patients with hemangioma in close proximity to major vascular structures. Possible reasons include the following: First, the robotic surgery system provides a stable and three-dimensional view with magnification of the field of operation. The high-quality images allow surgeons to detect the plane between the hemangioma and normal liver parenchyma and to identify the blood vessels supplying the hemangioma more easily. Second, the robotic surgery system is particularly applicable to liver parenchymal transection through a curved parenchymal plane. The combination of the EndoWristed Maryland bipolar forceps and a harmonic scalpel facilitates enucleation of liver hemangioma. Third, the EndoWristed technology with seven degrees of freedom allows rapid and precise suturing of vascular branches coming from the major vascular structures to the hemangioma.

When hepatic hemangiomas are located in posterosuperior liver segments, the difficulty and risks of surgery increase exponentially [41]. With the accumulation of experience, improvements in surgical skills and the development of new instruments, resection of posterosuperior segments using minimally invasive techniques has been shown currently to be feasible and safe [42, 43]. Whether robotic surgery used in the resection of posterosuperior segments can provide better perioperative outcomes than LLR is still controversial [44–47].

Surgical treatment for large liver hemangiomas carries a significant risk of massive intraoperative bleeding. The Memorial Sloan Kettering Cancer Center reported 52 patients who underwent open liver resection or enucleation for giant liver hemangioma of > 10 cm. Ten (19.2%) patients experienced blood loss of ≥ 1 L, and the median blood loss was 400 mL (range 17–10,000 mL) [8]. In our study, 116 (40.4%) patients had a giant liver hemangioma greater than 10 cm in diameter. The median blood loss was only 200 ml (range 50–2500 mL), which was significantly lower than that reported for open liver resection [38, 48, 49]. Our results also showed that the size, location, and the relationship of liver hemangiomas to major blood vessels were associated with intraoperative blood loss. These results are consistent with the results reported by other authors [50, 51].

A recent systematic review on robotic liver resections that included 31 studies and 1148 patients showed the overall complication and conversion rates to be 17.6% and 5.9% respectively [52]. Our study showed a nonsignificant difference in the overall postoperative complication rates between the robotic and laparoscopic groups (11.6% vs. 23.3%). However, all the Clavien‒Dindo grade II and III complications occurred in the laparoscopic group, a result possibly related to the increased surgical precision of using the robot which also shortened the postoperative hospital stay. In a retrospective analysis of the 2016–2018 Nationwide Readmissions Database by Aziz et al. [53], the robotic group showed a significantly lower complication rate than the laparoscopic or open group. A recent meta-analysis comparing robotic versus laparoscopic hepatectomy for HCC demonstrated that the rate of major complications was significantly lower in RALR than in LLR [54].

The rate of conversion to open surgery has been used as an indicator of technical feasibility [55]. Stiles et al. [55] reported a conversion rate of 19.1% for 1062 minimally invasive hepatectomies that were entered into the ACS‐NSQIP database from 2014 to 2015. This high conversion rate is probably attributed to the inclusion of inexperienced and small‐volume centers. The low conversion rate of 8% in our study was mainly related to all of the operations being performed by hepatobiliary surgeons with substantial experience in minimally invasive surgery working in a high-volume liver center. Our results showed that RALR had a tendency for a lower conversion rate than LLR (4.7% vs. 10.5%). Although no significant differences in the conversion rates have been reported between RALR and LLR [37, 56], a recent systematic review and two international multicenter retrospective analyses showed a significant reduction of conversion in RALR when compared with LLR [57–59]. Similar results were also reported by Fagenson et al. by using the NSQIP database [60].

There are several limitations of the study. First, this is a single-center retrospective comparative study with its inherent defects. The PSM method was employed to reduce any potential biases that may exist between the two groups. Second, perioperative outcomes are primarily dependent on the experience of the operating surgeons. To reduce this confounding factor, the operations in this study were performed by several surgeons who were experienced in open, robotic and laparoscopic hepatectomies. Third, there was a small sample size on patients who underwent RALR, thus lowering the statistical power. The potential type 2 errors due to the small sample size could not be obviated. However, to our knowledge, this study is the largest study directly comparing laparoscopic with robotic liver resection for hemangiomas ever reported in the medical literature. In the future, well-designed multicenter randomized studies comparing RALR with LLR for liver hemangioma are warranted.

In conclusion, this study showed that both RALR and LLR were safe and feasible in well-selected patients. RALR resulted in a longer operative time but a shorter postsurgical hospital stay. There were no significant differences in blood loss, conversion rate or postoperative overall morbidity rates between the two techniques. Hemangiomas located in posterosuperior liver segments and in close proximity to major vascular structures were independent predictors of increased intraoperative blood loss. For patients with liver hemangioma in close proximity to major vascular structures, robotic-assisted liver resection was better than laparoscopic liver resection.
